# Exploring the Link Between Caffeine Intake, Sleep Quality, and Restless Legs Syndrome Among Medical Students

**DOI:** 10.7759/cureus.84401

**Published:** 2025-05-19

**Authors:** Bhavna Singla, Syed Fakhar Abbas, Marium Nadeem Khan, Muneeba Amir, Muhammad Usairam Cheema, Muhammad Armaghan Akhlaq, Muhammad Abdullah Khan, Maheen Ahmed, Warda Mirza, Mazhar Ali, Sher Bano

**Affiliations:** 1 Internal Medicine, ECMC (Erie County Medical Center) Hospital, Buffalo, USA; 2 Neurology, Federal Government Polyclinic Hospital, Islamabad, PAK; 3 Internal Medicine, Shifa College of Medicine, Islamabad, PAK; 4 Internal Medicine, Foundation University Medical College, Islamabad, PAK; 5 General Internal Medicine, Services Institute of Medical Sciences, Lahore, PAK; 6 Otolaryngology, Services Institute of Medical Sciences, Lahore, PAK; 7 General Medicine, King's Mill Hospital, Sutton-In-Ashfield, GBR; 8 Internal Medicine, Dow Medical University, Karachi, PAK; 9 Internal Medicine, Capital Medical University, Beijing, CHN; 10 Internal Medicine, Khyber Medical College, Peshawar, PAK; 11 Clinical Psychology, NeuroWave Research Center, Islamabad, PAK

**Keywords:** caffeine consumption, lifestyle factors, medical students, restless legs syndrome (rls), sleep quality

## Abstract

Background

Medical students face increased threats to their sleep quality and lifestyle health because academic stress and unpredictable class hours create these challenges. Medical students commonly use too much caffeine as a stress management tool, but research lacks data about how caffeine affects both sleep quality and restless legs syndrome (RLS) symptoms.

Methods

The study utilized a research design combining cross-sectional analysis and correlation assessment with a participant group of 300 medical students ranging from 18 to 30 years old. Data was collected from January to March 2025. The Caffeine Consumption Questionnaire, combined with the Pittsburgh Sleep Quality Index (PSQI) and Restless Legs Syndrome Symptom Severity Scale, enabled researchers to collect necessary data. IBM SPSS Statistics for Windows, version 26 (IBM Corp., Armonk, NY) served as the platform to perform all statistical assessments through descriptive statistics, ANOVA tests, chi-square tests, correlation analysis, and multiple regression procedures.

Results

The medical students among the surveyed sample of 300 showed 237 participants (79%) caffeine consumption, while 78 participants (26%) consumed it nightly. Studies revealed that RLS symptoms affected 34 participants (11.2%) of participants as increased caffeine consumption resulted in milder RLS (r = -0.383, p < 0.01) yet negatively impacted sleep quality (r = 0.197, p < 0.01). The students who consumed caffeine in the morning experienced the highest sleep quality and the lowest severity of RLS symptoms (r = -0.266, p < 0.01). Additionally, poor sleep deteriorated RLS symptoms.

Conclusion

Higher caffeine use minimizes the reported intensity of RLS symptoms but leads to diminished sleep quality, particularly when coffee is taken late in the day. The combination of poor sleep and RLS symptoms creates a continuous worsening effect that persists across the cycle. Medical students' overall health and academic performance depend on specific intervention methods that teach proper sleep habits and caffeine consumption.

## Introduction

Sleep quality that depends on physiological and psychological, and environmental factors profoundly affects health because poor sleep results in fatigue and irritability, and compromised functioning [1]. Sleep quality refers to how contented someone feels about their restful periods, as well as sleep onset and physical restoration, which are quantified through personal logs known as sleep diaries [2]. A review of research findings from 57 studies demonstrated that medical students displayed poor sleep quality, to the extent of 52.7%, with specific regions showing higher rates, particularly in Europe and the Americas [3]. Therefore, standard screenings and intervention plans should be implemented [3].

Medical students displayed significant amounts of internet addiction and poor sleep quality during the COVID-19 pandemic [4]. Better sleep hygiene and coping strategies must become important parts of academic support because medical students develop poor sleep quality from excessive stress [5,6].

People commonly use coffee caffeine as a beverage, which affects their performance and sleep quality positively and negatively [7]. Medical students consume more caffeine during exam times and experience two main side effects of nervousness and sleeplessness [8]. The association between higher caffeine intake and poor sleep quality existed for non-evening drinkers exclusively, while chronotype failed to affect this connection [9]. Medical students showed increased consumption of caffeine, which correlated with increased anxiety levels and stress and deteriorated sleep quality [10,11].

The sensory neurological disorder, restless legs syndrome (RLS), produces day and night movements coupled with increased sleeplessness and poor mood states while affecting quality of daily life, although commonly tied to blood iron deficiency and heart diseases [12]. RLS affects 8% of young medical students, where the condition primarily affects women and leads to modest sleep disturbances, while smoking acts as a worsening factor [13]. A high prevalence of both poor sleep quality and RLS was detected among 6th-year medical students and residents, while multiple risk factors were identified [14].

RLS symptoms were correlated with stress, anxiety, and sleep disturbances in medical students. They could be related to functional impairment and daytime dysfunctionality, causing negative effects on academic performance, while insufficient RLS education demonstrated the necessity for enhanced patient screening [15]. Among medical students, 11.2% reported RLS; yet this condition deteriorated during periods of stress and examinations, leading to poor sleep outcomes [16]. RLS, which affects medical students with high frequency, shows a direct relationship to sleep issues, daytime sleepiness, drinking caffeine, as well as smoking habits, and existing medical conditions [17].

There are numerous studies conducted on the role of caffeine in sleep disturbances and RLS symptoms, but there is still a dearth of studies that particularly target medical students who are at risk group with high academic stress, irregular sleep patterns, and high caffeine intake. This research was designed to address that gap by investigating the cumulative effects of caffeine intake on sleep quality and the RLS symptomatology in this unique population group.

The combination of academic pressures and irregular class hours, along with psychological stress, creates a risk environment for medical students to experience lifestyle-related health issues. Students commonly use caffeine as a coping strategy to fight sleepiness and boost their concentration while simultaneously fighting tiredness.

The sleep disorder RLS usually receives little recognition among young adults. The neurological condition called RLS creates rest-related leg movement urges that disrupt sleep cycles while causing major life quality reductions. While the effects of caffeine on such neurological conditions as RLS have been studied in the general population, there has been relatively little research into this correlation within the medical student population, with these students being especially susceptible because of their high caffeine consumption and sleep disturbance.

The current investigation serves an essential purpose within the present research timeline. The study intends to address a critical literary deficiency through the investigation of how caffeine intake affects sleep quality, together with RLS symptoms in medical student populations. Knowledge about such connections educates us about the effects of everyday habits on students' health while aiding the creation of specific medical institution wellness initiatives. The research findings will enable medical institutions to develop student wellness initiatives that integrate both sleep hygiene counseling and caffeine regulation, which will advance academic results and total student wellness.

Objectives

This study aimed to assess the rate of caffeine consumption among medical students and evaluate their sleep quality regarding caffeine use. It also sought to investigate both the frequency and intensity of RLS symptoms within this population. Furthermore, the study aimed to explore the connection between caffeine consumption and RLS symptoms, as well as determine the association between sleep quality and the occurrence of RLS symptoms among medical students.

## Materials and methods

Study design and setting

This research used a cross-sectional correlational design to investigate the connections between medical students' caffeine consumption, together with their sleep quality and RLS symptoms. Students between the ages of 18-30 years, studying medicine, participated in the study. The investigators gave orientation and counseling to the students before engaging them and described study objectives, procedures, and confidentiality procedures. While performing data collection, strict measures were established to guarantee the quality of the data gathered. All the identified discrepancies in the data were successfully recorded and handled through established protocols. Missing values were managed using multiple imputation approaches, consistent with the data management procedures of the study to minimize bias and ensure the robustness of findings. Consent was obtained from those who consumed caffeine at least three times per week. Students with RLS diagnosis or other significant sleep disorders and pregnancy, alongside severe neurological or psychiatric conditions, were excluded from the study. Students taking medications that disturb regular sleep were excluded from the study, as well as students not enrolled in the medical courses. Termination of potential predictors, confounders, and effect modifiers such as tobacco smoking status, proton pump inhibitors (PPI) use, and alcohol exposure was not evaluated in this study, and this is recognized as a limitation. These factors, however, could not be controlled by statistical analysis. Future research should consider such variables to explain possible effects they may have on sleep quality and associated outcomes.

Data was collected from January to March 2025, including undergraduate medical students across all academic years. Medical students in this selected environment served as an appropriate research group because they commonly face elevated academic pressure along with disrupted sleep habits and increased caffeine intake, which affect sleep quality and possibly trigger RLS symptoms.

Sampling technique and sample size

The convenience sampling method was used because of its practical and logistical restrictions. The target population of this study was specific: medical students whose academic setting was limited. Thus, it was challenging to apply a probability sampling technique such as random or stratified sampling. With the restrictions of time, availability, and voluntary participation in the study, a convenience sampling yielded a sample who could be recruited efficiently, who were readily available, and who agreed to participate.​​​​​​ The sample size was computed utilizing the WHO sample size formula with a 95% confidence interval (CI), an estimated population proportion of 0.90, and a desired absolute precision of 0.05, which was calculated for 300 participants.

Data collection tools and procedure

Data for this research were gathered with three main instruments: the Caffeine Consumption Questionnaire, the Pittsburgh Sleep Quality Index (PSQI), and the Restless Leg Syndrome (RLS) Questionnaire. The Caffeine Consumption Questionnaire is a measure used to evaluate the amount, frequency, and timing of caffeine consumption in participants. It is designed to collect self-reported information about the participants' caffeine consumption habits, including the sources of caffeinated beverages (e.g., coffee, tea, energy drinks, or sodas), the frequency of consumption (e.g., daily, weekly), and the time of day. It was developed by Shohet and Landrum in 2001 [18]. We used the Pittsburgh Sleep Quality Index (PSQI) to get a comprehensive measure of sleep quality over the past month. PSQI was developed by Buysse et al. in 1989 [19]. The PSQI is a self-rated scale consisting of 19 items that generate seven component scores, which are then summed to determine a global score ranging from 0 to 21, where higher scores indicate worse sleep quality. PSQI is rated from 0 = no difficulty to 3 = severe difficulty and has a good internal reliability of a = .83 [19]. The Restless Leg Syndrome Questionnaire enables patients to measure their RLS symptoms through a self-administered survey that evaluates symptom occurrence and intensity. The Restless Legs Syndrome Symptom Severity Scale was developed in 2003 by Walters et al. [20]. The questionnaire consists of 10 validated items that healthcare providers or patients can use to report RLS severity through a rating system from 0 to 4 that measures symptoms, including discomfort, along with the urge to move, sleep disturbance, mood effects, and functional limitations. The assessment combines ten items into an overall score between 0 and 40 to measure symptom intensity. It has good internal consistency reliability (α=0.81, 0.80, and 0.76, respectively) [20]. A directed survey obtained participant information about age, gender, educational attainment, occupational status, marital status, and residential area. The data collection instrument consisted of Google Forms (Google LLC, Mountain View, CA) which participants used to respond.

Participating individuals received 20-30 minutes to finish all three surveys. All participants provided their data anonymously, while the research team ensured a guarantee of information privacy. All participants learned that joining the research was completely voluntary while retaining their right to stop involvement at any moment without enduring adverse effects. The study participants were 18 years or older, so there was no need for parental consent. The study received ethical approval from the institutional review board, which guaranteed that research procedures met all ethical requirements when working with human participants.

The Institutional Review Board, NeuroWave Research Center, Islamabad, Pakistan, granted approval (Approval No. IRB-2025-0017) before starting the research. The research evaluation used IBM SPSS Statistics for Windows, version 26 (IBM Corp., Armonk, NY) for its execution. Descriptive statistical analysis of participant characteristics and study variables employed frequencies, percentages, means, and standard deviations. The research used both independent samples t-tests together with one-way ANOVA to evaluate differences between groups based on variables including gender, the academic year, as well as sleep duration, and time of caffeine ingestion. Multiple linear regression tested the prediction strength of RLS between caffeine use and sleep quality. Chi-square tests evaluated the associations between categorical day-to-day demographic variables, which included age groups and academic classification, as well as caffeine consumption timeframes and duration of sleep patterns. The study considered a p-value less than 0.05 to indicate statistical significance.

## Results

Table 1 shows the distribution of demographics among 300 participants. Among the participants, 151 (50%) were aged 21-23 years, while 105 (35%) respondents self-identified as 18-20 years old. Participants aged 24-26 were 31 (10%), and only 13 (4%) were 27 or older. The study participants comprised 165 (55%) female students, while 135 (45%) were male. The study participants' majority belonged to their 3rd year of study (123 (41%) while 2nd-year students came next with 69 participants (23%) followed by 1st-year (45 {15%}), 4th-year (36 {12%}), and 5th-year (9 {3%}) students and house officers (15 (5%). Among the participants, 102 (34%) reported consuming one daily caffeine serving, but only 93 (31%) reported having two servings daily, and another 42 (14%) chose three servings. At the same time, 63 (21%) did not have any caffeine during the day. Among the participants, 81 (27%) initiated their caffeine consumption during the afternoon, 79 individuals (26.3%) consumed it at night, 76 individuals (25.3%) did not use caffeine at all, and only 64 participants (21.3%) consumed it in the morning. Of the participants surveyed about their nighttime sleep duration during weekdays, 96 individuals (32%) maintained a pattern of six to eight hours, 93 (31%) slept four to six hours, 63 (21%) received less than four hours of sleep, and 51 (17%) exceeded eight hours of sleep. The study results indicated that 204 individuals (68%) of participants were taking medications that influenced their sleep or alertness. The research data showed that 177 (59%) participants had family members suffering from RLS, while 90 (30%) participants did not experience this condition, and 36 (12%) participants were unaware if their family members had RLS.

**Table 1:** Demographic characteristics of participants (N=300) f=frequency, %=percentage

Variable	f	%
Age	-	-
18-20 years	105	35
21-23 years	151	50
24-26 years	31	10
27 years or more	13	4
Gender	-	-
Male	135	45
Female	165	55
Year of study	-	-
1st year	45	15
2nd year	70	23
3rd year	124	41
4th year	37	12
5th year	10	3
House officer	14	5
Daily caffeine intake	-	-
1 serving per day	103	34
2 servings per day	93	31
3 servings per day	42	14
I do not consume caffeine	62	21
Time of first caffeine intake	-	-
Morning (before noon)	64	21
Afternoon (12 pm – 6 pm)	81	27
Night (after 6 pm)	79	26
I do not consume caffeine	76	25
Duration of sleep on weekdays	-	-
Less than 4 hours	63	21
4-6 hours	92	31
6-8 hours	95	32
More than 8 hours	50	17
Use of medication affecting sleep or alertness	-	-
Yes	203	68
No	97	32
Family history of restless legs syndrome	-	-
Yes	176	59
No	89	30
Not sure	35	12

Table 2 displays the intercorrelations between the study variables. Research indicates that increasing caffeine levels in patients decreases the symptom severity of RLS (r = -0.383, p < 0.01). According to the PSQI (r = 0.197, p < 0.01), other research revealed that higher caffeine intake is statistically linked to worse sleep quality scores. The relationship between RLS and sleep quality was negative with a statistically significant value (r = -0.266, p < 0.01), which shows that worse RLS symptoms lead to poorer sleep quality.

**Table 2:** Intercorrelations between the study variables ** p<0.01 considered significant

Variable	1	2
Caffeine Consumption Questionnaire	-	-
Restless Legs Syndrome	-0.383^**^	-
Pittsburgh Sleep Quality Index	0.197^**^	-0.266^**^

Table 3 compares the mean scores of study variables across different age groups. Age groups differed significantly in caffeine consumption, F (3,296) = 2.666, p < 0.01, since students between 21 and 23 years consumed the most (M = 400.3, SD = 410.9), yet those between 24 and 26 years drank the least (M = 245.3, SD = 315.9). The youngest population group, between 18 and 20 years old, scored the highest on RLS measurements (F (3,296) = 6.772, p < 0.01). Participants aged 27 years or above recorded the lowest RLS scores (M = 26.2, SD = 2.0). Sleep quality showed no significant differences between age groups since F (3,296) = 0.866 remained non-significant. The practical effect of these measurements (η²) demonstrated minor statistical significance.

**Table 3:** Comparison of variables (age) M: mean, SD: standard deviation, F: F-ratio, η2: effect size ** p<0.01 considered significant

Variable	18-20 years	21-23 years	24-26 years	27 years or more	F (3,296)	η2
	M±SD	M±SD	M±SD	M±SD		
Caffeine Consumption Questionnaire	295.6±368.4	400.3±410.9	245.3±315.9	471.8±490.5	2.666^**^	0.0024
Restless Legs Syndrome	30.1±3.4	28.9±3.5	28.8±2.0	26.2±2.0	6.772^**^	0.0061
Pittsburgh Sleep Quality Index	47.7±10.3	47.9±4.0	48.1±5.5	51.1±8.1	0.866^**^	0.0008

Table 4 demonstrates how variables differ between students at diverse academic levels. Research revealed that students from the 3rd year consumed the most caffeine at M = 504.1 (SD = 412.4), followed by 1st-year students at M = 128.9 (SD = 177.4), F (5,294) = 8.962, p < 0.01. Research participants scored their RLS symptoms in different ways according to their study year, demonstrating significant differences both statistically F (5,294) = 9.956, p < 0.01 and numerically between 2nd-year students (M = 30.5, SD = 3.3) at one end of the scale and house officers (M = 27.1, SD = 3.2) at the other end. Students in their 3rd year produced the lowest measurements of sleep quality (M = 49.5, SD = 8.9) compared to other years, F (5,294) = 3.330, p < 0.01. Study results indicate a moderate practical relationship between student year classification and RLS scores, shown by an effect size of η² = 0.145.

**Table 4:** Comparison of variables (year of study) M: mean, SD: standard deviation, F: F-ratio, η2: effect size ** p<0.01 considered significant

Variable	1st year	2nd year	3rd year	4th year	5th year	House officer	F (5,294)	η2
	M±SD	M±SD	M±SD	M±SD	M±SD	M±SD		
Caffeine Consumption Questionnaire	128.9±177.4	234.6±338.0	504.1±412.4	346.3±416.8	260.2±376.8	363.4±453.7	8.962**	0.132
Restless Legs Syndrome	31.0±3.8	30.5±3.3	28.2±2.8	28.7±2.5	28.0±4.0	27.1±3.2	9.956**	0.145
Pittsburgh Sleep Quality Index	46.1±4.6	46.2±4.5	49.5±8.9	48.5±4.2	45.7±8.7	50.6±8.1	3.330**	0.054

Table 5 shows the results that compare individuals based on their sleep durations during weekdays. Participants who slept fewer than four hours or more than eight hours used more caffeine than those who slept between four and eight hours, as indicated by F (3,296) = 3.408 and p < 0.01. These individuals exhibited caffeine consumption levels of M = 444.4, SD = 422.8, and M = 433.9, SD = 412.9, respectively. Sleep duration between four and eight hours demonstrated higher RLS scores than other sleep patterns, as confirmed through a statistical test with F (3,296) = 4.763 and p < 0.01. The results showed no significant difference between conditions regarding sleep quality, as indicated by F (3,296) = 0.108. All effect sizes measured small values, indicating that the observable difference effects were minimal between groups, resulting in statistically significant changes.

**Table 5:** Comparison of variables (duration of sleep on week days) M: mean, SD: standard deviation, F: F-ratio, η2: effect size ** p<0.01 considered significant

Variable	Less than 4 hours	4-6 hours	6-8 hours	More than 8 hours	F (3,296)	η2
	M±SD	M±SD	M±SD	M±SD		
Caffeine Consumption Questionnaire	444.4±422.8	271.7±341.4	321.5±398.9	433.9±412.9	3.408^**^	0.003
Restless Legs Syndrome	28.3±3.4	29.6±2.5	29.8±3.3	28.2±2.5	4.763^**^	0.004
Pittsburgh Sleep Quality Index	47.8±6.9	48.1±10.4	47.8±4.5	48.5±3.4	0.108^**^	0.001

Table 6 shows the results of the variables measured when participants first started consuming caffeine. The data showed a significant difference in caffeine consumption, where F (3,296) = 4.829 indicated p < 0.01. People who took caffeine during the morning hours before noon recorded the highest caffeine consumption levels with a mean of 499.9 (standard deviation of 436.2). On the other hand, participants who started their caffeine intake in the afternoon between 12 and 6 pm showed the lowest levels of caffeine intake at 264.8 (standard deviation of 358.0). Caffeine users who started their consumption during the morning experienced the least RLS symptoms, according to analysis revealed via F (3,296) = 4.109, p < 0.01, and the results indicate low scores with M = 27.9 and SD = 2.7. The participants who consumed caffeine at different times demonstrated varying sleep quality levels according to F (3,296) = 1.926, p < 0.01, but had a minor practical influence with effect size (η² = 0.001). The participants who avoided caffeine showed average scores for the analysis variables.

**Table 6:** Comparison of variables (time of first caffeine intake) M: mean, SD: standard deviation, F: F-ratio, η2: effect size ** p<0.01 considered significant

Variable	Morning (before noon)	Afternoon (12 pm - 6 pm)	Night (after 6 pm)	I do not consume caffeine	F (3,296)	η2
	M±SD	M±SD	M±SD	M±SD		
Caffeine Consumption Questionnaire	499.9±436.2	264.8±358.0	308.5±383.1	360.7±377.6	4.829^**^	0.004
Restless Legs Syndrome	27.9±2.7	29.7±3.7	29.6±3.3	29.1±3.2	4.109^**^	0.003
Pittsburgh Sleep Quality Index	49.0±3.3	45=6.4±6.7	48.3±11.1	48.5±3.8	1.926^**^	0.001

Table 7 presents findings from a multiple regression analysis that evaluates RLS through the variables, including caffeine consumption and sleep quality. The results showed that higher caffeine consumption significantly predicts RLS with a standardized coefficient of β = -0.344, t = -3.81, p < 0.01. The study findings imply that increased caffeine consumption tends to reduce RLS symptom levels, but these results require additional research to validate clinical implications. The PSQI results showed that inferior sleep quality was a noteworthy negative predictor of RLS symptoms with B = -0.093, 95% CI (-0.142, -0.044), β = -0.199, p < 0.01.

**Table 7:** Multiple regression for study variables B: coefficient, SE: standard error, β: standardized coefficient, LL: lower limit, UL: upper limit, Cl: confidence interval ** p<0.01 considered significant

Variable	B	95% Cl		SE	β
		LL	UL		
Constant	34.650	32.3	36.9	1.19	-
Caffeine Consumption Questionnaire	-.003	-.004	-.002	.000	-.344^**^
Pittsburgh Sleep Quality Index	-.093	-.142	-.044	.025	-.199^**^

Table 8 shows that participants were distributed according to their ages throughout their academic years. The 18-20-year-old participants (n = 105, 35%) showed a preference for the 2nd (n = 37, 35%) and 3rd years (n = 39, 37%). Participants who were aged 21-23 years enrolled most in the 3rd year (73, 48%), yet their numbers were split between the 2nd year (30, 20%) and 4th year (22, 15%) respectively. The distribution of people aged 24-26 years (n = 31, 10%) was split between the 3rd (n = 12, 39%) and 4th years (n = 9, 29%). Of the participants aged 27 years or older (n = 13, 4%), house officers (n = 11, 85%) were the predominant type. Statistical results from the chi-square test indicated a significant relationship between subject year and participant age group (χ² = 234.3, p = 0.000), which confirms a strong connection between these variables.

**Table 8:** Descriptive statistics of demographic variables (age and year of study) f: frequency, %: percentage, p: level of significance p-values calculated using the chi-square test, the significance level is set at p < 0.05

Variable	f	1st year (%)	2nd year (%)	3rd year (%)	4th year (%)	5th year (%)	House officer (%)	P-value	χ²
Age	-	-	-	-	-	-	-	-	-
18–20 years	105	19 (18%)	37 (35%)	39 (37%)	6 (6%)	2 (2%)	2 (2%)	0.000	234.3
21–23 years	151	23 (15%)	30 (20%)	73 (48%)	22 (15%)	3 (2%)	0 (0.0%)	–	–
24–26 years	31	3 (10%)	3 (10%)	12 (39%)	9 (29%)	3 (10%)	1 (3%)	–	–
27 years or more	13	0 (0%)	0 (0%)	0 (0%)	0 (0%)	2 (15%)	11 (85%)	–	–

Table 9 investigates the connection between participant age groups and sleep duration patterns. Most of the young adults between 18 and 20 years of age (n = 105) spent nightly durations ranging from four to six hours (n = 37, 35%) and six to eight hours (n = 35, 33%). Participants between 21 and 23 years old (n = 151) mostly rested between six and eight hours (n = 48, 32%) or four and six hours (n = 47, 31%). People between 24 and 26 years of age (n = 31) indicated two matching rates of approximately 29% (n = 9) who slept six to eight hours and 29% (n = 9) who slept less than four hours. A split distribution of sleep durations occurred equally among the 27 or older participants, who totaled 13 individuals. The chi-square statistics revealed that age did not affect sleep duration patterns since the results showed χ² = 12.9 and a p-value of 0.153.

**Table 9:** Descriptive statistics of demographic variables (age and sleep duration) f: frequency, %: percentage, p: level of significance p-values calculated using the chi-square test, the significance level is set at p < 0.05

Variable	f	<4 hours (%)	4–6 hours (%)	6–8 hours (%)	>8 hours (%)	P-value	χ²
Age	-	-	-	-	-	-	-
18–20 years	105	13 (12%)	37 (35%)	35 (33%)	20 (19%)	0.153	12.9
21–23 years	151	36 (24%)	47 (31%)	48 (32%)	20 (13%)	–	–
24–26 years	31	9 (29%)	6 (19%)	9 (29%)	7 (23%)	–	–
27 years or more	13	5 (39%)	2 (15%)	3 (23%)	3 (23%)	–	–

Table 10 demonstrates the time when students started consuming caffeine during their different academic years. Most individuals who consumed caffeine during their morning hours belonged to the 3rd academic year (n = 31, 49%) and totaled 64 respondents. The 2nd- and 3rd-year students composed equally large groups of afternoon caffeine consumers, with each group containing 28 students (35%). Among the night-time caffeine consumers (n = 79), there was a maximum enrollment in the 3rd academic year (n = 30; 38%). Data showed that many students who did not consume caffeine belonged to the 3rd year of their studies (n = 35, 46% of the total 76 students). Results from the chi-square test demonstrated academic level as a factor that shapes when students start consuming caffeine (χ² = 32.3, p = 0.006).

**Table 10:** Descriptive statistics of demographic variables (time of first caffeine intake and year of study) f: frequency, %: percentage, p: level of significance p-values calculated using the chi-square test, the significance level is set at p < 0.05

Variable	f	1st year (%)	2nd year (%)	3rd year (%)	4th year (%)	5th year (%)	House officer (%)	P-value	χ²
Time of first caffeine intake	-	-	-	-	-	-	-	-	-
Morning	64	5 (8%)	7 (11%)	31 (49%)	12 (19%)	3 (5%)	6 (10%)	0.006	32.3
Afternoon	81	12 (15%)	28 (35%)	28 (35%)	6 (7%)	5 (6%)	2 (3%)	–	–
Night	79	20 (25%)	16 (20%)	30 (38%)	9 (11%)	1 (1%)	3 (4%)	–	–
I don’t consume caffeine	76	8 (11%)	19 (25%)	35 (46%)	10 (13%)	1 (1%)	3 (4%)	–	–

Table 11 explores how the time of morning caffeine consumption relates to total nighttime sleep length. The sleep duration of six to eight hours, along with over eight hours, was the most prevalent among morning caffeine consumers, who total 64 (n = 30, 47% and n = 21, 33%). Most of the individuals who took caffeine in the afternoon (n = 81) either slept for less than four hours (n = 29, 36%) or four to six hours (n = 24, 30%). Most nighttime caffeine consumers experienced sleep lengths of fewer than four hours, along with durations of six to eight hours during their sleep periods. Survey respondents who did not drink caffeine mainly got sleep for four to six hours (n = 30, 40%) in addition to six to eight hours (n = 24, 32%). The analysis revealed that the time when participants first drank caffeine directly influenced their sleep patterns since these variables exhibited a significant connection (χ² = 48.3, p = 0.000). demonstrates the time when students started consuming caffeine during their different academic years. Most individuals who consumed caffeine during their morning hours belonged to the 3rd academic year (n = 31, 49%) and totaled 64 respondents. The 2nd- and 3rd-year students composed equally large groups of afternoon caffeine consumers, with each group containing 28 students (35%). Among the night-time caffeine consumers (n = 79), there was a maximum enrollment in the 3rd academic year (n = 30; 38%). Data showed that many students who did not consume caffeine belonged to the 3rd year of their studies (n = 35, 46% of the total 76 students). Results from the chi-square test demonstrated academic level as a factor that shapes when students start consuming caffeine (χ² = 32.3, p = 0.006).

**Table 11:** Descriptive statistics of demographic variables (time of first caffeine intake and sleep duration) f: frequency, %: percentage, p: level of significance p-values calculated using the chi-square test, the significance level is set at p < 0.05

Variable	f	<4 hours (%)	4–6 hours (%)	6–8 hours (%)	>8 hours (%)	P-value	χ²
Time of first caffeine intake	-	-	-	-	-	0.000	-
Morning	64	1 (2%)	12 (19%)	30 (47%)	21 (33%)	-	48.3
Afternoon	81	29 (36%)	24 (30%)	19 (24%)	9 (11%)	–	–
Night	79	16 (20%)	26 (33%)	22 (28%)	15 (19%)	–	–
I don’t consume caffeine	76	17 (22%)	30 (40%)	24 (32%)	5 (7%)	–	–

## Discussion

The research investigated the relationship patterns between medical students consuming caffeine as well as their RLS symptom severity, alongside sleep quality measurements. The participant sample mainly contained female subjects from an age range of 21-23 who were focusing on their 3rd academic year. Many participants in this sample group regularly consumed caffeine since their main consumption times occurred during the afternoons and evenings. Our research indicated that higher levels of caffeine consumption led to diminished RLS symptoms, together with worse sleep quality, according to the obtained statistical relations. Clinical research shows that caffeine elevates symptoms in RLS patients because it stimulates both the nervous system and the muscles while existing literature agrees that RLS negatively affects sleep quality. The disagreement between results about caffeine effects probably originates from differences in how people respond to caffeine and the doses that each individual takes [21]. Prior studies conducted in older adults also support these findings, which documented the worsening of sleep quality in association with greater RLS symptom severity and its negative effects on general well-being. Thus, our study reconfirms the previously noted inverse relationship between RLS symptoms and sleep quality [22].

The study data revealed that younger participants consumed more caffeine than older participants because research demonstrates age-related increases in caffeine consumption patterns. Numerous health problems, such as disrupted sleep, occur when young people consume high levels of caffeine [23]. Research indicates RLS shows high occurrence rates in teenagers who most often develop symptoms between puberty and early adulthood. Studies have already established that RLS develops most often during the young adult years, thus affecting their sleep habits and overall health status. The study results showed that younger participants had higher RLS scores, implying RLS could develop during early life before continuing through successive years, just like other cohort studies have found [24].

The results of our research matched earlier findings, demonstrating that 3rd-year students drank the most caffeine because their caffeine consumption increased during their educational progression. The tendency to consume more caffeine emerges during periods of increased academic pressure, which students encounter throughout their academic journey, particularly during examination times [25]. Research conducted on medical students established a connection between RLS symptoms and both caffeine consumption and sleep issues, together with feelings of tiredness. The study results match those obtained from our research [17]. The research data demonstrates that RLS symptoms reach their highest point during the second academic year among medical students.

The obtained data supports prior research linking caffeine consumption to insomnia symptoms while demonstrating different impacts that depend on one's average sleep hours. The results follow our findings because people with disrupted sleep patterns who consume more caffeine face additional sleep-related issues [26]. The REST (RLS Epidemiology, Symptoms, and Treatment) General Population Study documented the general RLS burden on sleep quality, but our study shows that people who sleep between four to eight hours experience the highest RLS severity compared to other sleep patterns [27]. Further studies should analyze how certain sleep duration intervals affect RLS symptom magnitude [27].

Our study revealed that participants who took their daily caffeine intake in the morning had decreased RLS scores and better sleep quality than those who consumed it later in the day. Research confirms that taking caffeine in the morning produces fewer sleep disturbances and better academic performance, thus supporting the present findings [28].

The research demonstrates a negative relationship between caffeine consumption and RLS symptoms, yet counter-evidence shows high levels of caffeine could worsen these symptoms in some instances [21]. The study revealed increased caffeine use as an RLS symptom-reducing factor and sleep quality deterioration as an RLS symptom-worsening factor. Studies have validated the relationship between Restless Legs Syndrome and disturbed sleep patterns, which matches our current research [29].

Previous research supports the discovery that student sleep durations are shortened as academic levels and age groups increase, which may result from academic pressures and lifestyle changes. Sleep duration varies based on the combination of student age together with academic responsibilities [30].

The research demonstrates that students between 18-20 years old consume caffeine throughout the morning and maintain steady sleep schedules because these groups follow established patterns of morning types who abstain from caffeine while achieving improved academic results. Students who begin their day with caffeine tend to sleep between six and eight hours because these factors create a connection between their academic level and their habit of using caffeine [31].

The study faces a major limitation due to its dependent nature on self-reported data because participants might incorrectly report their caffeine consumption along with their sleep disturbances. To minimize the chances of improper reporting, participants were given simple instructions supplemented by standardized examples for commonly consumed caffeinated drinks and sleep-related symptoms. The questionnaire was presented in a structured and easy-to-understand approach and anonymity was maintained to get genuine feedback. The cross-sectional nature of the research hinders conclusions about whether caffeine use causes poor sleep or RLS symptoms because it only presents information at a single point in time. This research examined only a single regional medical student cohort; therefore, it cannot be generalized to larger populations or different stress-facing student demographics.

More research should be conducted using long-term observation techniques like cohort studies or continuous sleep diaries that are kept for a period of over a few weeks or even months and controlled experiments such as the randomized trials investigating the effects of differing amounts of caffeine the quality of sleep has on individuals for purposes of ascertaining the correction relationship that exists between sleep and fatty deposits. The use of objective tools such as actigraphy and biochemical caffeine measurement would improve dataset accuracy. In-depth insights regarding the connections between dose-dependent caffeine effects and individual tolerance levels and psychological factors such as anxiety, depression, and RLS symptoms need further research. Research validity would increase if the study expanded to investigate multiple student populations at various institutions while identifying vulnerable groups.

## Conclusions

The research demonstrates how RLS symptoms, sleep quality, and caffeine ingestion affect medical students through multiple healthcare-related factors. Research shows that higher caffeine intake decreases RLS symptoms but simultaneously leads to inferior sleep quality, particularly when patients consume it later during the day. The study confirms disturbed sleep functions as a significant RLS symptom intensifier, especially when students in this population practice correct sleep habits. Younger students and those in the first stages of medical education presented higher RLS scores and caffeine consumption rates, thus showing they were more susceptible during their early academic years. The research underlines requirements for strategic academic programs to help promote better sleep techniques and coffee control in educational settings. Improved well-being and academic performance in future healthcare professionals will result from increased awareness about dangerous health risks related to excessive caffeine use and poor sleep quality.

## References

[1] Nelson KL, Davis JE, Corbett CF (2022). Sleep quality: an evolutionary concept analysis. Nurs Forum.

[2] Kline CE (2020). Sleep quality. Encyclopedia of Behavioral Medicine.

[3] Rao WW, Li W, Qi H (2020). Sleep quality in medical students: a comprehensive meta-analysis of observational studies. Sleep Breath.

[4] Tahir MJ, Malik NI, Ullah I (2021). Internet addiction and sleep quality among medical students during the COVID-19 pandemic: A multinational cross-sectional survey. PLoS One.

[5] Almojali AI, Almalki SA, Alothman AS, Masuadi EM, Alaqeel MK (2017). The prevalence and association of stress with sleep quality among medical students. J Epidemiol Glob Health.

[6] Al-Khani AM, Sarhandi MI, Zaghloul MS, Ewid M, Saquib N (2019). A cross-sectional survey on sleep quality, mental health, and academic performance among medical students in Saudi Arabia. BMC Res Notes.

[7] O'Callaghan F, Muurlink O, Reid N (2018). Effects of caffeine on sleep quality and daytime functioning. Risk Manag Healthc Policy.

[8] Lakshmi Devi SS, Abilash SC, Basalingappa S (2018). The rationale of caffeine consumption and its symptoms during preparatory and non-preparatory days: a study among medical students. Biomed Pharmacol J.

[9] Kerpershoek ML, Antypa N, Van den Berg JF (2018). Evening use of caffeine moderates the relationship between caffeine consumption and subjective sleep quality in students. J Sleep Res.

[10] Akova İ, Duman EN, Sahar AE, Sümer EH (2023). The relationship between caffeine consumption and depression, anxiety, stress level and sleep quality in medical students. J Turk Sleep Med.

[11] Nasir GM, Ahmad J, Aziz A, Hussain H, Zafar R, Iqbal A (2023). Effect of caffeine consumption on sleep quality of undergraduate medical students of Multan. J Fatima Jinnah Med Univ.

[12] Manconi M, Garcia-Borreguero D, Schormair B, Videnovic A, Berger K, Ferri R, Dauvilliers Y (2021). Restless legs syndrome. Nat Rev Dis Primers.

[13] Ishaq M, Riaz SU, Iqbal N (2020). Prevalence of restless legs syndrome among medical students of Karachi: an experience from a developing country. Sleep Disord.

[14] Ergin N, Kılıç BB, Ergin A, Varlı S (2022). Sleep quality and related factors including restless leg syndrome in medical students and residents in a Turkish university. Sleep Breath.

[15] Yıldız T, Kafadar D, Fenercioğlu AK, Şenel GB, Sipahioğlu NT (2024). Prevalence and awareness of restless legs syndrome and associated self reported sleep problems in medical students. J Turk Sleep Med.

[16] Al-Hunaiti T, Ashour L, Jamal B (2024). Restless legs syndrome and its associated factors among Jordanian medical students: a cross-sectional study. East Asian Arch Psychiatry.

[17] Alabdulqader R, Alqahtani R, Alsunaikh K, Almousa S, Alsalem A, Alokley A (2025). Prevalence and risk factors of restless legs syndrome among medical students in Saudi Arabia: an observational study. Ann Afr Med.

[18] Shohet KL, Landrum RE (2001). Caffeine consumption questionnaire: a standardized measure for caffeine consumption in undergraduate students. Psychol Rep.

[19] Buysse DJ, Reynolds CF 3rd, Monk TH, Berman SR, Kupfer DJ (1989). The Pittsburgh Sleep Quality Index: a new instrument for psychiatric practice and research. Psychiatry Res.

[20] Walters AS, LeBrocq C, Dhar A, Hening W, Rosen R, Allen RP, Trenkwalder C (2003). Validation of the International Restless Legs Syndrome Study Group rating scale for restless legs syndrome. Sleep Med.

[21] Lutz EG (1978). Restless legs, anxiety and caffeinism. J Clin Psychiatry.

[22] Cuellar NG, Strumpf NE, Ratcliffe SJ (2007). Symptoms of restless legs syndrome in older adults: outcomes on sleep quality, sleepiness, fatigue, depression, and quality of life. J Am Geriatr Soc.

[23] Soós R, Gyebrovszki Á, Tóth Á, Jeges S, Wilhelm M (2021). Effects of caffeine and caffeinated beverages in children, adolescents and young adults: short review. Int J Environ Res Public Health.

[24] Silva GE, Goodwin JL, Vana KD, Vasquez MM, Wilcox PG, Quan SF (2014). Restless legs syndrome, sleep, and quality of life among adolescents and young adults. J Clin Sleep Med.

[25] Lee KH, Human G, Fourie J, Louw W, Larson C, Joubert G (2009). Medical students’ use of caffeine for “academic purposes” and their knowledge of its benefits, side-effects and withdrawal symptoms. South Afr Fam Pract.

[26] Chaudhary NS, Grandner MA, Jackson NJ, Chakravorty S (2016). Caffeine consumption, insomnia, and sleep duration: results from a nationally representative sample. Nutrition.

[27] Allen RP, Walters AS, Montplaisir J, Hening W, Myers A, Bell TJ, Ferini-Strambi L (2005). Restless legs syndrome prevalence and impact: REST general population study. Arch Intern Med.

[28] Mitchell PJ, Redman JR (1992). Effects of caffeine, time of day and user history on study-related performance. Psychopharmacology (Berl).

[29] Moreira NC, Damasceno RS, Medeiros CA, Bruin PF, Teixeira CA, Horta WG, Bruin VM (2008). Restless leg syndrome, sleep quality and fatigue in multiple sclerosis patients. Braz J Med Biol Res.

[30] Kohyama J, Ono M, Anzai Y (2020). Factors associated with sleep duration among pupils. Pediatr Int.

[31] Cole JS (2015). A survey of college-bound high school graduates regarding circadian preference, caffeine use, and academic performance. Sleep Breath.

